# “If I can get my work done, I’m not addicted to my phone”: Investigating smartphone use among language students

**DOI:** 10.1371/journal.pone.0342041

**Published:** 2026-05-05

**Authors:** Qian Wang, Kizito Tekwa

**Affiliations:** 1 Department of Basic Education, Hefei Technology College, Hefei, Anhui, China; 2 Shenzhen Technology University, Shenzhen, China; Prague University of Economics and Business: Vysoka Skola Ekonomicka v Praze, CZECHIA

## Abstract

The study of smartphone addiction among college students reveals significant gaps, particularly in understanding its impact on specific academic groups, such as language learners, whose unique reliance on mobile applications (MAs) for learning remains underexplored. Existing research, often limited by cross-sectional designs and self-reported data, lacks context-specific insights into sociocultural factors and personality influences. This study addresses these lacunae by examining MA usage among 31 third-year English majors at a Chinese university perceived to be addicted based on the Smartphone Addiction Scale-Short Version (SAS-SV). The purpose was to investigate usage patterns, their correlation with academic performance and personality traits, and whether high usage amounted to perceived addiction within this cohort. A mixed-methods approach was employed, using 2 weeks of objective app-tracking data, a 50-item personality questionnaire, academic performance metrics, and qualitative reflections on usage motivations. Findings revealed 150,551 minutes spent on top MAs (WeChat, Xiaohongshu, Douyin), with 83.9% denying addiction despite high use, while 16.1% admitted compulsive behaviors affecting sleep and academics. Conscientiousness negatively correlated with usage time. No other correlations were found. Qualitatively, students cited communication, study, and entertainment as key drivers. Implications suggest reframing addiction as “high but largely functional smartphone engagement,” while advocating for policies to curb compulsive usage identified in 16.1% of participants. Universities should implement usage guidelines and provide counseling to foster a balanced digital environment.

## 1. Introduction

Smartphone addiction among college students has emerged as a pressing concern in the digital age, with excessive mobile application (MA) use increasingly linked to academic, psychological, and physical challenges. Studies indicate a rising prevalence, particularly in Asia, where Lyu et al. reported a significant increase in Mobile Phone Addiction Index (MPAI) scores among Chinese college students, from 36.55 in 2013 to 46.25 in 2022 [1]. This addiction, characterized by compulsive usage, salience over other activities, and negative consequences [2], has been associated with anxiety (r = 0.37), depression (r = 0.42), and reduced academic performance [3]. Social media and communication apps, such as WeChat and Douyin, dominate usage, often blurring the line between productive and problematic engagement. However, the lack of consensus on diagnostic criteria, as noted in the DSM-5’s absence of smartphone addiction classification, complicates its study, with prevalence estimates varying widely due to reliance on self-reports [4].

Despite this growing body of research, significant lacunae persist. Most studies focus on broad student populations, overlooking specific groups such as language students, whose curricular demands (e.g., vocabulary apps like Duolingo) may uniquely shape their MA usage patterns [5]. The reliance on cross-sectional designs limits causal inferences about addiction’s impact [3], while the lack of objective data and standardized measures, as critiqued by Panova and Carbonnel [6], undermines validity. Sociocultural contexts, such as academic pressure in non-Western settings like China, remain underexplored, and the relationship between personality traits and MA use in language-learning contexts is poorly understood [1]. These gaps hinder the development of tailored interventions for mobile-assisted learning.

This study aims to bridge these lacunae by examining MA usage among 31 third-year English majors at a Chinese university, all self-identified as addicted phone users. It addresses three research questions: 1) What are the patterns of MA usage (time and frequency) and their correlation with academic performance? 2) How do personality traits (e.g., extraversion, neuroticism) relate to MA usage? 3) What are the reasons for MA use, and how do they shape addiction perceptions and academic engagement? By focusing on a specific cohort, the study employs a mixed-methods approach, using two weeks of objective app-tracking data (verified via peer-checked spreadsheets), a 50-point personality questionnaire, academic performance metrics, and qualitative reflections on usage motivations.

The significance of this study lies in its contribution to filling research gaps with robust, context-specific data, offering insights into how language students’ unique digital behaviors impact learning. The study provides a foundation for developing targeted pedagogical strategies and policies to harness technology’s benefits while mitigating addiction risks in higher education.

## 2. Literature review

### 2.1. Definition and conceptualization of smartphone addiction

Smartphone addiction, often termed problematic smartphone use, is characterized by excessive, compulsive engagement with mobile applications (MAs), leading to significant functional impairment or distress [6]. Core symptoms include recurrent failure to control usage, salience (prioritizing phone use over other activities), withdrawal symptoms, and persistence despite negative consequences [7–10] emphasize two key components: significant impairment and persistence over time. However, defining smartphone addiction remains contentious due to its overlap with behavioral addictions rather than substance dependence. Panova and Carbonell [6] argue that smartphone use often lacks the severe physical dependence and relapse patterns seen in substance addictions, suggesting the term “problematic use” may be more appropriate. The absence of standardized diagnostic criteria in the DSM-5 and the variability of screening tools further complicate the construct’s validity, leading to inconsistent prevalence rates and potential over-pathologization [4,6]. This definitional ambiguity underscores the need for precise frameworks to study specific populations, such as language students with self-reported app addiction.

Chinese studies often use terms such as mobile phone addiction, mobile phone addiction tendency, mobile phone dependence, or problematic mobile phone use, but they describe a similar underlying construct, i.e., an obsessive or dependent state in which individuals experience psychological, physiological, and social dysfunction when they cannot use their phones [11,12]. Conceptualizations commonly emphasize loss of control, withdrawal symptoms (irritability, anxiety, restlessness when separated from the phone), craving, and the use of phones to regulate negative emotions such as boredom, loneliness, or stress [13,14].

Smartphone addiction is rarely treated as a formal psychiatric diagnosis; instead, it is framed as a dimensional tendency or problematic behavior that can reach clinically significant levels. Authors frequently highlight that it is not yet recognized in DSM-5 or ICD-11, but argue that its functional impact—sleep problems, academic failure, and mental health difficulties—warrants serious attention [13].

Several themes are routinely studied alongside smartphone addiction. Academic and learning outcomes are a central focus, with studies linking higher addiction scores to greater academic procrastination, reduced learning engagement, and poorer grades [11,12,15]. Mental health variables such as depression, anxiety, social anxiety, loneliness, and stress are also repeatedly associated with higher addiction levels [13,14]. Sleep quality and physical health complaints, including insomnia, fatigue, and autonomic symptoms, are another prominent cluster of correlates [13]. Finally, self-control, time management, and personality traits (e.g., shyness) are often examined as risk or protective factors shaping the development and severity of mobile phone addiction [15,12,14].

### 2.2. Current studies and findings on smartphone addiction among students

Research on smartphone addiction among college students has grown, with a focus on its prevalence and impact on academic and psychological outcomes. Studies indicate a significant rise in addiction levels, particularly in Asian countries like South Korea and China [1,5]. For instance, Lyu et al. (2024) found that Mobile Phone Addiction Index (MPAI) scores among Chinese college students increased from 36.55 in 2013 to 46.25 in 2022, with a large effect size (Cohen’s d = 0.88). Common predictors include negative emotions (e.g., anxiety, r = 0.37; depression, r = 0.42), low social support (r = −0.12), and lack of resilience (r = −0.13) [16] reported weak-to-moderate correlations between mobile phone addiction (MPA) and psychological issues among college students, including anxiety (r = 0.39), depression (r = 0.36), impulsivity (r = 0.38), and poor sleep quality (r = 0.28). Achangwa et al. [3] highlighted adverse effects in South Korean university students, such as increased stress, reduced academic performance, and physical health issues like musculoskeletal pain and sleep disorders. Al-Barashdi et al. [17]noted that 25.8% of Jordanian university students exhibited addiction, with negative impacts on grades and focus. Notably, no studies have explored smartphone addiction among language students, despite their unique academic demands, such as engagement with language-learning apps, which may interact distinctly with problematic usage patterns [5].

Research across China consistently demonstrates that elevated levels of smartphone or mobile phone addiction are strongly associated with adverse academic, psychological, and health outcomes among students. In academic contexts, mobile phone addiction correlates with increased procrastination and diminished self-regulated learning and time management [15]. Among university and medical students, higher addiction scores are predictive of reduced learning engagement, poorer perceived academic performance, and heightened academic burnout [11,18]. These patterns underscore smartphone addiction as a detrimental influence on academic functioning within China’s higher education landscape, where intense educational pressures, such as prolonged medical training and declining graduate employment rates, often drive students to use smartphones as a form of escapism, further exacerbating disengagement from learning [19,20]. Notably, students constitute the largest segment of netizens in China, accounting for 26.9% of users, which amplifies the linkage between addiction and academic impairment [20].

Psychologically, mobile phone addiction frequently coexists with traits such as shyness, social anxiety, loneliness, and low self-control [14,18]. Evidence indicates that shyness or social anxiety elevates addiction risk, while low self-control acts as a critical mediator or amplifier of problematic usage [11,14]. Addiction is further tied to increased depressive symptoms and emotional distress [13,18]. These associations are particularly pronounced in China’s collectivist cultural framework, where conformity motives, such as the desire to fit in socially, are more salient, prompting greater reliance on smartphones for interpersonal connections [21]. Historical policies like the pre-2016 one-child mandate may have diminished traditional social support networks, heightening this dependency and contributing to loneliness [22]. Drawing on Self-Determination Theory, addiction can be framed as stemming from low intrinsic motivation in a high-pressure society, where interdependent social structures emphasize the role of support in mitigating psychological vulnerabilities [20,22]. The COVID-19 lockdowns intensified these dynamics, amplifying reliance on digital ties for identity formation among adolescents amid restrictions on family and school interactions [21,23].

Regarding physical health and sleep, higher addiction levels are linked to autonomic symptoms, including palpitations and somatic discomfort, alongside poorer sleep quality, extended sleep latency, and reduced sleep duration, effects that are especially evident when daily smartphone use surpasses several hours [13]. Investigations employing graded addiction categories (none, mild, moderate, severe) reveal dose-response relationships, with escalating severity corresponding to progressively worse psychological, physical, and sleep outcomes [13]. However, definitions of addiction in these studies eschew strict hourly thresholds, prioritizing symptomatic indicators such as dependency, withdrawal, and functional impairment over mere quantity of use. Implicitly, addiction involves a “constant increase in time” spent online, leading to discomfort without access and distraction from real-life responsibilities [20]. Contextual references suggest “overuse” might imply more than 4–6 hours per day in Chinese settings, though this is not a formal cutoff; for instance, students engaging in “considerable time” on non-academic virtual activities heightens risk [24]. Behavioral analyses emphasize time composition, such as reallocating 30–60 minutes from sedentary screen time to moderate-to-vigorous physical activity to lower addiction susceptibility, yet diagnostic scales focus on uncontrollable patterns rather than hours alone [20]. Thresholds remain variable, with unendorsed alignments to guidelines like the World Health Organization’s recommendation of under 2 hours of daily recreational screen time for youth, underscoring that addiction is defined by its uncontrollable nature and resultant harms rather than duration per se.

The research also highlights substantial heterogeneity in the mechanisms and consequences of addiction across subgroups, influenced by China’s diverse socio-economic and regional contexts. Among medical students, problematic mobile phone use is robustly connected to academic burnout, diminished learning dedication, inferior academic performance, and intensified psychological distress, encompassing depression, social anxiety, loneliness, and sleep disruptions [18]. In this demanding milieu, characterized by rapid economic growth and ubiquitous smartphone access, devices initially function as maladaptive stress-coping tools but evolve into compulsive habits that sustain cycles of fatigue and impairment [24]. Regional variations further illuminate these dynamics: in capitalist, Western-influenced Hong Kong, where universities primarily enroll local students, addiction predictors center on entertainment and information seeking; conversely, in socialist mainland areas like Guangzhou, with diverse student populations from across China, social interaction emerges as a dominant factor, reflecting broader societal shifts toward digital ubiquity [24].

By contrast, physical education majors and general undergraduates manifest addiction chiefly through procrastination, eroded self-regulated learning, and time management deficits [15,11]. Structural equation modeling reveals that mobile phone dependence, both directly and indirectly, fosters procrastination by undermining academic self-efficacy and temporal orientation. Psychologically vulnerable students face amplified risks, leveraging smartphones for compensatory social engagement and emotion regulation [11,12,14], while regular exercise bolsters self-control and attenuates addiction [12]. High-intensity users and those with sedentary lifestyles encounter addiction via circadian disturbances, prolonged sleep latency, and compromised sleep quality, which intensify daytime impairments and perpetuate nighttime usage [11,13]. Differences across academic years and leadership involvement suggest that structured offline activities provide some safeguards [18]. Collectively, these findings advocate for culturally attuned interventions, such as bolstering professional identity in medical training, to address addiction within China’s evolving educational and digital environment.

### 2.3. Limitations and avenues for future research

Despite these insights, current research has significant limitations. First, the reliance on cross-sectional designs limits causal inferences about whether smartphone addiction drives academic or psychological declines or vice versa [3,4]. Second, many studies rely on self-report measures, which introduce bias and inflate prevalence rates due to discrepancies between perceived and actual use [6,16]. Third, the focus on broad student populations overlooks specific academic groups, such as language students, whose curricula may uniquely influence app usage [1,5]. Fourth, the lack of standardized diagnostic criteria and the diversity of measurement instruments undermine construct validity and comparability across studies [4]. Finally, sociocultural and contextual factors, such as academic pressure or cultural norms around technology use, are underexplored, particularly in non-Western settings [6,17].

According to researchers, these gaps can be bridged by pursuing several avenues. Longitudinal studies are needed to establish causality by tracking how problematic app use affects academic outcomes over time, especially among language students [3]. Employing diverse, validated instruments (e.g., the Smartphone Addiction Scale) and objective data (e.g., app-tracking software) can enhance measurement accuracy [16]. Qualitative approaches, such as interviews, could uncover motivations for app use in specific contexts, such as language learning [2]. Investigating specific app types (e.g., social media vs. educational apps) and their differential impacts on academic performance is crucial, particularly for language students who may rely on specialized apps [4]. Finally, exploring psychological and sociocultural factors, such as anxiety or peer dynamics, in underrepresented groups, such as language students, can provide tailored insights for mobile-assisted learning interventions [1,17]. These avenues will advance a more nuanced understanding of smartphone addiction in educational settings.

### 2.4. How this study bridges current gaps in the literature

This study, examining mobile application (MA) usage among 31 third-year English majors with self-reported app addiction at a Chinese university, bridges critical gaps in the literature on smartphone addiction in educational contexts. By focusing on language students, a demographic unexplored in prior research [1,5], it addresses the lack of targeted studies on specific academic groups. Unlike broad surveys often criticized for their generality [25], this study’s mixed-methods approach, combining objective app usage tracking, personality trait assessments, and qualitative reflections, provides nuanced insights into the behaviors of a high-risk cohort. The use of device-tracked data, verified through peer-reviewed spreadsheets, mitigates reliance on self-report measures, a noted limitation in the field [6,16]. By analyzing two controlled weeks of data, the study minimizes confounding variables, enhancing the validity of findings on app usage patterns and their impact on learning. The inclusion of personality traits, assessed via a 50-point questionnaire, responds to calls to explore psychological factors like [4]. Furthermore, qualitative reports on students’ reasons for app use and preferences for curriculum integration fill gaps in understanding user motivations, which were often speculative in prior studies [17]. This study sets the pace for research on language students and employs robust methods to enrich the discourse on mobile-assisted learning and app addiction in higher education. It answers the following questions:

1) What are the patterns of MA usage (time and frequency) among these students, and how do these correlate with their academic performance and personality traits?
2) What are the reasons these students use specific MAs, and how do these reasons relate to their academic engagement and performance?

These questions address literature gaps by targeting language students, an understudied group, and linking objective app usage data to academic performance, mitigating reliance on self-reports [1,6], exploring psychological factors like personality traits [4], and qualitatively investigating motivations and academic impacts to enhance understanding of user behaviors in specific academic contexts [3,17].

## 3. Methodology

This study, conducted during the 18-week winter semester at a Chinese university, spanned 8 weeks to explore mobile application (MA) use among 31 third-year English majors perceived as smartphone addicts. The methodology details the mixed-methods approach employed to answer the three research questions. This section outlines the participants, data collection, instruments, and data analysis.

### 3.1. Participants

The participants (28 females, 3 males) were enrolled in the Educational Technologies course, which provided students with theoretical and practical knowledge on the role of technology in language learning. In addition to their core English courses, they studied Culture Studies, Business English, and other languages (Spanish, French, Japanese) to diversify their knowledge. Students were recruited in the third week after the course introduction. Thirty-three of the 38 students volunteered to participate in the study. Participants completed a screening survey, adapted from the Smartphone Addiction Scale-Short Version (SAS-SV) [26], to confirm problematic app usage indicative of addiction. Two participants were excluded due to incomplete device-tracked data (three days instead of seven). All participants used smartphones that tracked daily screen time and app usage. They agreed to record weekly MA data on an online spreadsheet 10 minutes before Thursday classes (covering Friday to Wednesday) and complete Thursday’s data on Friday mornings. The research was presented neutrally during recruitment in the Educational Technologies course as an exploration of “mobile application usage patterns among language students,” with no explicit mention of “smartphone addiction” or related pathological terms to avoid priming biased self-presentations. Participants volunteered based on this description, and the subsequent screening survey, adapted from the Smartphone Addiction Scale-Short Version (SAS-SV), was positioned as a general assessment of “app usage habits” rather than an addiction diagnostic. Ethical approval was obtained from the educational institution. Additionally, all participants provided informed consent prior to the commencement of the study.

### 3.2. Data collection and instruments

Data collection occurred over eight weeks, with two consecutive weeks (Weeks 4 and 5) analyzed to minimize confounding variables (e.g., exams, extracurricular activities). At the study’s outset, participants completed a 50-item personality trait questionnaire [27] to assess extraversion, neuroticism, openness, conscientiousness, and agreeableness. A WeChat group was created to facilitate communication between participants and the instructor. The sample size is consistent with current research on mobile-assisted language learning [28].

Twice a week (Thursday and Friday mornings), participants manually entered device-tracked MA data (total screen time, top three most frequently used applications [MFAs], and individual MA usage time [MAUT]) into a shared spreadsheet. To ensure data accuracy, students worked in pairs, with each student verifying the data entered by their peers. Additionally, students were asked to update their personal notes to explain their usage during that particular period. The notes were expected to be made every day to coincide with data usage. They included explanations for MFAs each day. Academic performance data, specifically course grades, covered the four major assignments completed during the semester, with a focus on the month in which the data were collected. At the study’s conclusion, participants submitted 150-word reflective reports via email, detailing their MA usage patterns, self-perceived addiction, and how MA use affected their academic engagement and performance.

### 3.3. Data analysis

*RQ1: Patterns of MA usage and correlation with academic performance*. Research Question 1 examined patterns of mobile application usage time (MAUT) and frequency (MAUF) and their relationship with academic performance. Total MAUT was calculated by summing minutes spent on all tracked applications across the two-week period, separately for Week 1, Week 2, and overall. MAUF was calculated by counting how many times each application was opened. Descriptive statistics (means, standard deviations, ranges, and individual rankings) were computed for total MAUT, the three most frequently used applications per participant, and category-level usage. To test differences in MAUT between Week 1 and Week 2, paired-samples t-tests were conducted. One-way ANOVA was used to compare time spent on the first-, second-, and third-most accessed applications. Pearson product-moment correlations were calculated to examine relationships between MAUT (overall and weekly), time spent on educational applications, and academic performance (monthly and overall course grades). All analyses were performed using IBM SPSS Statistics Version 28, with statistical significance set at p < .05.

Pearson’s correlation coefficients (r) were calculated to examine bivariate relationships between MAUT and MAUF and personality traits. Correlation strength was interpreted following Cohen’s [29] conventions: |r| ≈ .10 (small), |.30| (medium), and |.50| (large).

*RQ2: Reasons for MA use and relation to academic engagement and performance.* NVivo was used to analyze reflective reports and daily notes, coding reasons for MA use under themes (e.g., entertainment, study, social interaction). Sub-themes were ranked into ten categories to identify usage patterns. Responses linking MA use to academic engagement (e.g., class participation) and performance (e.g., grades, assignment quality) were coded to assess impacts, with findings presented as percentages and thematic summaries.

### 3.4. Ethical considerations

This study was conducted in accordance with the requirements of the Helsinki Declaration and with ethical approval from the Department of Basic Education, Hefei Technology College (Approval No. DBE1012242, of October 12, 2024).

Written informed consent was obtained from all participants between October 18 and 20, 2024.

## 4. Findings

### 4.1. RQ1: MAUT and MAUF and correlation with academic performance

#### 4.1.1. How much time was spent on MAs collectively and individually?.

Participants spent 151262 minutes (i.e., 2521 hours 02 minutes) on MAs, including 150551 minutes (2509 hours 11 minutes) in the three MFAs. That means their MAUT was 77498 and 73053 minutes, respectively, in Week 1 and Week 2. In other words, they spent 1291 hours and 38 minutes in Week 1 and 1,217 hours and 33 minutes in Week 2. Because all participants took the same number of lessons per week, we did not believe this difference was due to course load. Further analysis based on MAUT per day showed that although more time was spent in Week 1, one day in Week 2 had more MAUT than any day in Week 1, as indicated in Table 1.

**Table 1:** MAUT data in Week 1 and Week 2.

	Week 1		Week 2		Difference between Week 1 and Week 2	
	Min	Hr/Min	Min	Hr/Min	Min	Hr/Min
Day 1 (Friday)	12518	208 hours and 38 minutes	11639	193 hours and 59 minutes	879	14 hours and 39 minutes
Day 2 (Saturday)	11742	195 hours and 42 minutes	10821	180 hours and 21 minutes	921	15 hours and 21 minutes
Day 3 (Sunday)	11208	186 hours and 48 minutes	11165	186 hours and 5 minutes	43	43 minutes
Day 4 (Monday)	10127	168 hours and 47 minutes	10649	177 hours and 41 minutes	−522	− 8 hours and 42 minutes
Day 5 (Tuesday)	10997	183 hours and 17 minutes	9356	155 hours and 56 minutes	1641	27 hours and 21 minutes
Day 6 (Wednesday)	10931	182 hours and 11 minutes	9639	160 hours and 39 minutes	1292	21 hours and 32 minutes
Day 7 (Thursday)	9975	166 hours and 15 minutes	9784	163 hours and 4 minutes	191	3 hours and 11 minutes
Total	**77498**	**1,291 hours and 38 minutes**	**73053**	**1,217 hours and 33 minutes**	**4445**	**74 hours and 5 minutes**

We computed the data into SPSS to determine whether there were statistical and practical differences between the datasets. Our analysis failed to confirm a difference between Week 1 (M = 11071.14; SD = 883.20) and Week 2 (M = 10436.14; SD = 856.25), p = .07 (non-significant). The absence of statistical differences between the two weeks indicates that participants’ overall MAUT did not change significantly, despite differences in the numerical values.

We compared weekdays and weekends and found a higher MAUT on Saturday and Sunday (M = 11234.00; SD = 380.38) than on weekdays (M = 10561.50; SD = 986.81); however, no statistical differences were found in the datasets, meaning that MAUT did not depend on whether they were at home or in school or whether they had lessons or not. Given that there were no overall differences, we investigated whether individual differences could reveal more nuanced behavioral patterns or preferences.

**Individually,** we analyzed MAUT for the most frequently used applications (MFAs) to determine whether the same behavioral pattern was evident. Our analysis confirmed the differences in MAUT in the two weeks, with more time spent on MAs in Week 1 (M = 2542.16; SD = 650.32) than in Week 2 (M = 2356.55; SD = 543.41). Furthermore, we found statistical differences between the two weeks’ data, with a p-value of.002 and a large effect size (Cohen’s d = 3.10).

**Fig 1:**
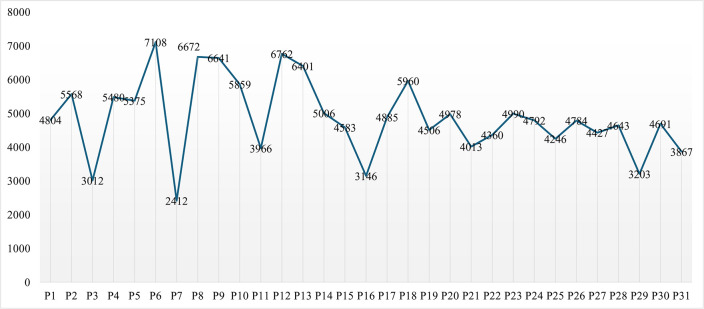
Total time spent on MAs by participants.

It was found that the lowest time spent on MA over the two weeks was 2412 minutes (40 hours 12 minutes), while the highest was 7108 minutes (118 hours 28 minutes), as indicated in Fig 1.

The data show that P7 spent the least time on MAs, while P6 spent the most. According to the data, participants with the highest MAUT included P2, P4, P5, P6, P8, P10, P12, P13, P14, and P18, who spent over 5000 minutes on MAs in two weeks, i.e., 83 minutes and 20 minutes on MAs. The descriptive statistics showed a mean of M = 4898.71 and a standard deviation of SD = 1157.69.

**Fig 2:**
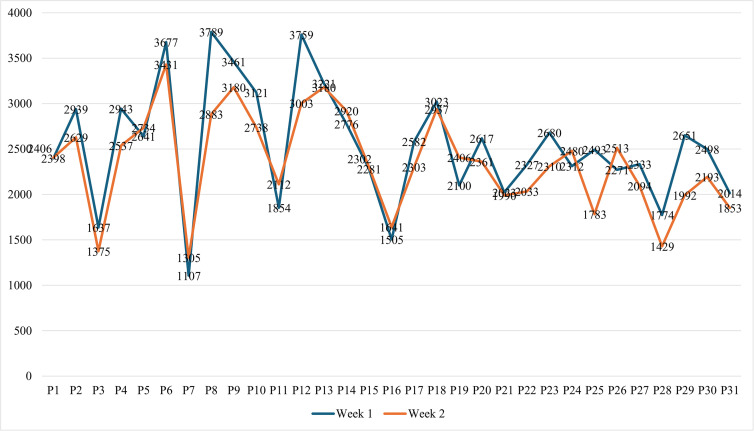
Individual MAUT for the three most frequently used apps.

Regarding the weekly MAUT, we found that more time was spent in Week 1 than in Week 2, confirming the pattern observed across all MAs over the two weeks. Our analysis indicated that the lowest and highest MAUT in Week 1 were 1107 minutes (18 hours 27 minutes) and 3789 minutes (63 hours 09 minutes), respectively. In Week 2, the minimum MAUT was 1305 minutes (21 hours 45 minutes), and the maximum was 3003 minutes (50 hours 03 minutes), as indicated in Fig 2.

The data also showed that most participants had more MAUT in Week 1. However, a significant number (10 participants) communicated more in Week 2, including P1 (−8minutes), P5 (−93minues), P7 (−198 minutes), P11 (−258 minutes), P14 (−144 minutes), P15 (−21 minutes), P16 (−136 minutes), P19 (−306 minutes), P24 (−168 minutes), and P26 (−242 minutes). Accordingly, P19 had the most significant negative MAUT difference in Week 2, while P8 (+906 minutes) had the most significant positive MAUT difference, followed by P12 (+756 minutes) and P25 (+710 minutes). We analyzed the information on whether students perceived themselves as addicted to their phones. The analysis showed that the majority, i.e., 90% (n = 28), believed they were not addicted, while the minority, i.e., 10% (n = 3), claimed to be “a little addicted.”

Our total weekly MAUT data showed no statistical differences; however, individual MAUT showed significant differences in the two weeks’ data. Given that participants took the same course and had the same number of lessons, we presumed that differences in MAUT could be due to variations in individual behavior, preferences, and usage patterns. Therefore, we investigated the MFAs before analyzing differences in participants’ usage patterns.

**Perception of addiction:** Based on the data, students were expected to make a case for their phone addiction. While 26 (83.9%) of the students did not believe they were addicted, 5 (16.1%) thought they were addicted. However, when we attempted to associate perceptions on addiction with other data, we found no correlations with MAUT, the three most accessed MAs, personality traits, and performance. We also found no correlations with the number of educational apps accessed. The absence of associations suggests that perceptions of addiction are not driven by these variables.

### 4.1.2. Which were the most frequented applications (MFAs)?

During the two weeks, participants accessed 81 MAs, including 56 in Week 1 and 58 in Week 2. By application, our MAUF data showed that WeChat MAs ranked highest, followed by Xiaohongshu and Weibo. We found that WeChat was by far the most popular MFA, used by almost every participant, and ranked as their most popular MA. However, when we calculated frequency based on time spent on the applications (i.e., MAUT), we found that WeChat still ranked first, followed by Xiaohongshu and Douyin, as outlined in Table 2.

**Table 2:** List of 15 MFAs based on accessed times and duration.

Rank	App Name	Times Used	Total time (mins)	Total time (hrs/mins)
1	WeChat	323	78512	1308 hrs 32 mins
2	Xiaohongshu	187	19367	322 hrs 47 mins
3	Weibo	158	7662	127 hrs 42 mins
4	Douyin	90	11850	197 hrs 30 mins
5	Bilibili	69	9172	152 hrs 52 mins
6	Baicizhan	43	104	1 hrs 44 mins
7	Taobao	33	1382	23 hrs 2 mins
8	Books	29	47	47 mins
9	Shanbei Word App	28	1649	27 hrs 29 mins
10	Duolingo	26	178	2 hrs 58 mins
11	Camera	25	707	11 hrs 47 mins
12	Quark	18	3443	57 hrs 23 mins
13	知乎 (Zihu)	17	2532	42 hrs 12 mins
14	WPS	17	228	3 hrs 48 mins
15	Identity Five	13	1964	32 hrs 44 mins

The data showed that WeChat and Xiaohongshu were the most consistent, ranking first and second in MAUT and MAUF, respectively. Weibo ranked third based on MAUF but behind Douyin and Bilibili in terms of MAUT. Also, although Baicizhan and Books were accessed 43 and 29 times, the total time spent on them was only 104 and 47 minutes, respectively, meaning the MAs ranked last in terms of MAUT. Similarly, though 知乎 (Zhihu), a Chinese question-and-answer platform similar to Quora, was accessed only 17 times, users spent 42 hours and 12 minutes on it, indicating that, in terms of MAUT, the MA ranked higher than most MAs in Table 2. Despite these individual differences, our data analysis showed a strong correlation between MAUT and MAUF (r(81)=.91, p = .001).

**Fig 3:**
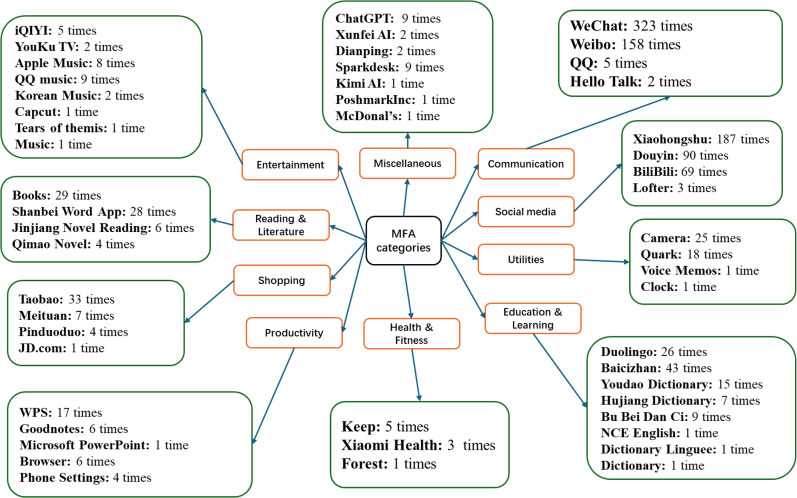
MA use categories.

We ranked the MFAs into 10 categories, as indicated in Fig 3, and analyzed the number of applications and the time spent in each category.

According to the data, the MFAs were in the entertainment, education and learning, and productivity categories, with each comprising five or more applications.

In total, the MAs were accessed 1,178 times (Communication = 497; Social Media = 428; Education and Learning = 81; Reading and Literature = 70; Shopping = 45; Utilities = 50; Productivity = 34; Entertainment = 27; Health and Fitness = 9; and Miscellaneous = 9). The data indicate that the volume of MAs in each category did not always translate to the frequency of access. In particular, although more MAs were consulted in the entertainment category, students spent less time on those MAs than on MAs in the communication, social media, education and learning, reading and literature, shopping, utilities, and productivity categories. The data shows a strong preference for communication, social media presence, education and learning, and reading. In contrast, there is a low preference for health and fitness, entertainment, productivity, utility, and shopping.

Additionally, we found that 90586, 39109, and 20856 minutes were spent on the top three MFAs, respectively. That means the second-ranked MAs were accessed less than half as often as the top-ranked MAs. For its part, the third-ranking MA was accessed significantly less than the preceding two types. We analyzed the data in SPSS to confirm the pattern and frequency using a one-way ANOVA. The results showed statistical differences (*p* = .001) among the three datasets, with a moderately large effect size (Cohen’s *d* = .65).

**Fig 4:**
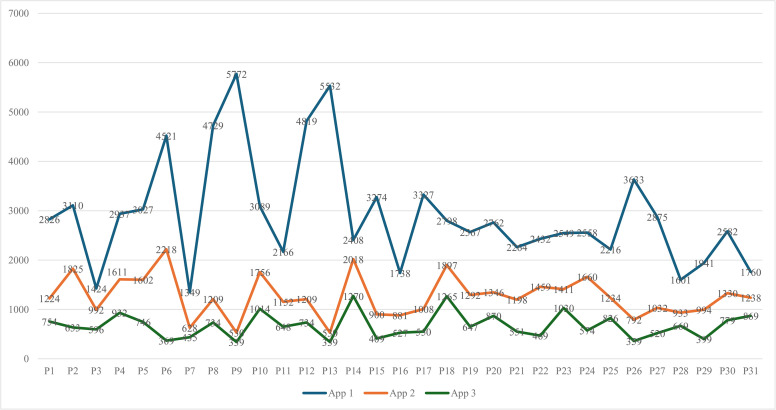
MAUT for the top three MFAs per participant.

Individually, there were considerable variations in usage among students for each app. For example, P6 spent 4, 521 minutes (75hrs 21mins) on their MFAs, while P7 spent only 1,349 minutes (i.e., 22hrs 29 mins). These differences were noticed for individuals and for each week. for App 1, while P7 has only 1,349. As presented in Fig 4, there were differences in MAUT for the top three MFAs for each participant.

For App 2 (those that ranked second among the three MFAs), we found that P6, for instance, spent 2218 minutes on MFAs in this category, while P9 only spent 530 minutes. The same pattern appears in App 3, where P31 spent 869 minutes, while P26 spent only 359. The data indicated that students had preferences for different MAs and they spent unequal amounts of time on them, likely based on individual needs or preferences.

### 4.1.2. Relation between MAUT and academic performance and personality traits

To investigate the relationships among Mobile Application Usage Time (MAUT), time spent on educational applications, and academic performance, Pearson product–moment correlations were conducted on a sample of 31 students (*N* = 31). MAUT was measured in Weeks 1 and 2; time spent on educational apps was recorded in total minutes; and academic performance was assessed both for the month of data collection and overall course performance. All correlations used a two-tailed test of significance.

MAUT for Week 1 was strongly positively correlated with total minutes spent on educational apps, *r*(29) =.76, *p* < .001, indicating that students who spent more time on mobile applications during Week 1 also devoted more time to educational apps. Similarly, MAUT for Week 2 showed a strong positive correlation with time on educational apps, *r*(29) =.68, *p* < .001, suggesting consistent patterns of app usage across the two weeks.

In contrast, MAUT showed no relationship with academic performance. For Week 1, MAUT was not significantly correlated with either monthly academic performance, *r*(29) =.07, *p* = .73, or overall course performance, *r*(29) =.07, *p* = .73. Likewise, MAUT for Week 2 showed weak, non-significant correlations with monthly performance, *r*(29) =.13, *p* = .47, and overall performance, *r*(29) =.13, *p* = .47. These findings suggest that time spent on mobile applications had little to no association with academic outcomes.

Time spent on educational apps also showed no relationship with academic performance. Total minutes on educational apps did not correlate with either monthly performance, r(29) =.03, p = .86, or overall course performance, *r*(29) =.03, *p* = .86, indicating that increased time on educational apps did not correspond to improved academic outcomes.

Overall, these results demonstrate that while students who spent more time on mobile applications (MAUT) in Weeks 1 and 2 also tended to spend more time on educational apps, neither MAUT nor time on educational apps was significantly associated with academic performance, whether measured monthly or across the entire course.

**Correlation between personality traits:** Among the Big-Five traits measured, only Conscientiousness showed a strong negative association with total objective smartphone usage time (r = –.806, p < .001, N = 31); students scoring higher on Conscientiousness spent substantially less time on mobile applications over the two-week period. Extraversion, Agreeableness, Neuroticism, and Openness showed no significant relationships with MAUT or MAUF (all |r| < .30, all p > .10).

### 4.3. RQ3:MAUT explanation and perceptions on phone addiction

We analyzed the reasons students gave for choosing the three MFAs and grouped them into seven main uses and functions: communication, study and learning, entertainment and relaxation, information and updates, social networking and discovery, practical tools, and fitness and health. Simultaneously, we sought to understand why students’ perceptions of addiction were based on their MAUT.

#### 4.3.1. Reasons for phone use.

**Communication**: Students used MAs like WeChat to stay in touch with family, access important course information and deadlines via group chats, coordinate group assignments and share life updates, and benefit from other versatile features such as messaging, video calls, and payment options. Because WeChat has many functions, it was the go-to MA for most students.

**Study and learning:** Students used applications like Baidu Cloud, Quark, Baicizhan, Pagago, Duoliingo, Goodnotes, and Shanbei Word to store and organize study materials and access academic resources, look up and memorize foreign language vocabulary, learn foreign language in a fun and exciting way, take notes in class, and engage in self-learning.

**Entertainment and relaxation:** According to students’ notes, MAs such as Bilibili, Douyin, and iQIYI, as well as Qimao Novel, are classified under entertainment and relaxation. The MAs were accessed to obtain educational content, including tutorials and entertainment videos. Meanwhile, Douyin was mainly used to watch short videos and access personalized content recommended by the MA based on students’ viewing history. The MAs also allowed students to watch TV shows and movies or read novels for relaxation.

**Information and updates:** We found that social media sites like Xiaohongshu and Weibo were utilized mainly to stay abreast of news and current affairs. Furthermore, Xiaohongshu was used to access public examination tips, shopping advice, and lifestyle content from user-generated posts. It was also used as a search engine for various topics and interests, all related to lifestyle and current news. Weibo, in contrast, mainly enabled students to stay up to date on trending topics while following their favorite personalities (musicians, actors/actresses, TV personalities).

**Social networking and discovery:** Social networking MAs included Xiaohongshu, WeChat, and Weibo, and they were used to explore fashion, obtain beauty tips, and other lifestyle recommendations. In addition, they helped connect with a community of users who shared similar experiences and insights. They were useful for building networks with peers and young professionals, especially for students who had taken internships.

**Practical tools:** Our data analysis showed that students also used certain MAs for their practical functionality. For instance, among the MAs in this category, Camera, Voice Memos, Safari, Meituan, JD.com, and Ele.Me were used to capture important moments of their lives and take notes during lessons. Others were used to take snapshots of the instructors’ PPT during the leson. They also used some MAs to record assignments and lectures, browse the Internet for information on homework and other life-related topics, and order food when it was not convenient to dine in the school canteen.

**Fitness and health:** These MAs were used to help students stay fit and track their progress. Furthermore, they were used to access physical training courses and workout routines. This means users learned and practiced these routines through self-regulated learning.

#### 4.3.2. Perceptions on phone addiction.

The data (see Fig 4) showed the time spent on the three most accessed apps, with peaks at 5,732, 5,582, and 2,075 units, indicating significant phone engagement among students. Despite this, the majority denied addiction, offering diverse perceptions to justify their usage.

P1 confidently stated, “I use WeChat for group projects; it’s a necessity, not an addiction,” suggesting productivity drives their screen time. P5 reinforced this control, saying, “I can stop whenever I want; it’s not like I’m glued to it,” emphasizing self-discipline. Many viewed their habits as intentional, with P12 noting, “WeChat helps me with my studies—how can that be addiction?” This reflected a belief that educational or social apps legitimize extended use. P19 added context, “My phone use changes when I do different activities; it’s not a daily thing, and I still get everything done,” indicating situational peaks rather than constant reliance. P23 corroborated, saying, “I use WeChat to stay connected; everyone’s doing it,” aligning their behavior with peers.

These perceptions hinged on functionality over duration. P31 asserted, “If I still get my work done, I’m not addicted,” prioritizing academic success as a measure of control. P7 admitted, “Sometimes I scroll late, but it’s just relaxing,” hinting at potential overindulgence masked as leisure. P15 counters, “I set limits for myself; it’s all about balance,” showcasing a proactive stance.

This denial may stem from stigma or gaps in self-awareness. P25 reflected, “Addiction sounds extreme—my usage feels normal,” suggesting a disconnect between data and perception. While students frame their time as purposeful, the chart’s variability raises questions. Without deeper insight into life impacts, their justifications hold, though the line between habit and addiction remains blurry.

While most students denied addiction to their phones, a few openly admitted to struggling. These students offered candid insights into their experiences, shedding light on the challenges they face.

P3 confessed, “I think I am addicted to Douyin. I can’t stop checking it—it’s like a reflex,” indicating a compulsive pull that disrupts their day. P9 echoes this, saying, “Douyin keeps me up at night; I know it’s too much,” highlighting how usage interferes with sleep, a key concern per the American Psychiatric Association. This suggests that for some, the time spent transcends mere habit into dependency. P14 added, “I’ve missed assignments because I am on my phone; it’s a problem,” revealing academic impacts that align with addiction signs.

Some students recognized the emotional toll. P18 admitted, “I feel anxious if I’m not on my phone—it’s weird. I want to cut back, but I think it is hard to do.” This showed that some students lacked self-control despite being aware of their excessive phone use. Their honesty contrasted with peers’ denials, possibly stemming from self-reflection or external feedback, like parental concerns.

The data’s variability supports their claims, with frequent spikes suggesting entrenched patterns. Unlike others who justify usage, these students see it as a battle. P30 concludes, “I think I am addicted; I need to figure out how to control myself. This minority perspective underscored that, for some, the phone’s grip exceeds casual use, warranting closer attention to its role in their lives.

## 5. Discussion

The present study, employing objective two-week app-tracking data among 31 third-year English majors in China, revealed strikingly high levels of smartphone engagement (an average of approximately 5.8 hours per day per participant) yet uncovered no significant associations between total Mobile Application Usage Time (MAUT) and academic performance, and only weak, non-significant links with Big-Five personality traits (except a modest trend for extraversion predicting higher usage). Most strikingly, 83.9% of participants denied being addicted despite objective usage far exceeding the implicit 4–6-hour “overuse” thresholds commonly referenced in Chinese addiction research [24,20], while the remaining 16.1% openly acknowledged compulsive patterns that interfered with sleep and assignments.

These findings stand in marked contrast to the dominant narrative in Chinese smartphone addiction literature. Large-scale survey and meta-analytic studies consistently report moderate-to-strong negative correlations between addiction scores and learning engagement [20], self-control [22], self-esteem, social support, and academic outcomes [15,11,18], alongside positive associations with depression, anxiety, and burnout [13,14]. The absence of academic harm in the current sample challenges the assumption that high usage is inherently detrimental when it occurs in a subpopulation – foreign language majors – whose curriculum explicitly encourages or even requires mobile-assisted language learning (MALL). Apps such as Duolingo, Shanbei Word, Baicizhan, and Pagago were frequently accessed for vocabulary acquisition and self-study, and time spent on educational applications was strongly positively correlated with overall MAUT (r = .68–.76, p < .001). This suggests that, for language students, a substantial proportion of “addictive-looking” screen time may actually represent productive, curriculum-aligned behavior, thereby blunting the negative academic and psychological consequences observed in medical, nursing, or general undergraduate cohorts [19,18].

Culturally, the widespread denial of addiction despite objectively high usage reflects a distinct Chinese normalization process. Participants repeatedly framed intensive smartphone engagement as “necessary” for group coordination (WeChat), staying connected with family and peers (collectivist relational maintenance), conforming to classmates’ communication norms, and meeting curricular demands – justifications that echo Wen et al.’s [21] findings on stronger conformity and social-relationship motives among Chinese youth, and Ding et al.’s [22] observation that diminished offline social support (exacerbated by the former one-child policy) pushes reliance onto digital platforms. In a high-pressure, exam-oriented educational culture where “getting work done” is the ultimate metric of self-worth, students appear to apply a functional threshold for addiction: “If I can get my work done, I’m not addicted” – a pragmatic definition that diverges sharply from symptom-based clinical or research scales such as the SAS-SV or MPAI.

WeChat’s overwhelming dominance (1,308 collective hours in two weeks) further illustrates a uniquely Chinese ecosystem effect. Unlike Western contexts where multiple fragmented apps compete, WeChat functions as an “everything app” (communication, payments, academic group chats, mini-programs, file sharing), making prolonged sessions structurally unavoidable for university life. This platform’s multifunctionality blurs the boundary between “addictive” and “essential” use more than in any other national context, and likely contributes to both the elevated baseline usage and the perceptual decoupling of time from pathology observed here.

The lack of a weekday–weekend difference in MAUT also departs from patterns reported in broader Chinese student samples [23], possibly because language-learning activities (vocabulary apps, reading, listening practice) are distributed evenly across the week rather than being confined to leisure periods. Personality findings partially align with prior work – extraversion driving higher social-app usage mirrors Liang & Leung’s [24] social-interaction motive in mainland students – but the overall weak predictive power (contrary to the R^2^ = .705 regression in an earlier pilot analysis) reinforces mounting evidence that, once objective multifunctional use is controlled for, traditional Big-Five traits explain less variance in Chinese student populations than previously assumed.

Overall, these results strongly support calls to move away from universal “addiction” labels toward a more nuanced concept of “problematic versus functional high engagement” [6]. For language majors in China, intensive smartphone use appears to have shifted from a risk factor to an adaptive academic strategy, at least in the short term, as measured by grades. This subpopulation-specific pattern underscores the danger of applying general addiction models to culturally and pedagogically distinct groups.

Future research should therefore adopt longitudinal designs with objective tracking to establish whether today’s “functional high users” eventually develop tolerance, withdrawal, or academic decline, or whether MALL integration creates a genuinely sustainable digital habit. Intervention efforts in Chinese universities may be more effective if they target the small minority who already exhibit clear compulsive symptoms (sleep interference, missed deadlines) rather than attempting blanket screen-time reduction, and if they leverage existing platforms (e.g., WeChat mini-programs for digital wellness or spaced-repetition vocabulary tools) instead of fighting against them.

In conclusion, the present findings reveal that smartphone “addiction” in China is not a monolithic phenomenon but is profoundly shaped by academic discipline, platform ecology, and cultural definitions of functionality. Language students’ ability to sustain very high levels of smartphone use without apparent academic or psychological costs challenges alarmist narratives and highlights the need for context-sensitive, objective, and culturally grounded approaches to smartphone research and policy in Chinese higher education. Institutions may seek ways to manage and support app usage patterns, ensuring tools like WeChat and Duolingo enhance learning without overwhelming students. Additionally, staff training to recognize signs of problematic use and to offer counseling services can address the 16.1% at risk. Universities must proactively shape a technology-friendly yet health-conscious academic environment that emphasizes using apps for learning rather than entertainment.

## 6. Conclusion

The field of addiction studies has long grappled with lacunae, particularly in understanding high but largely functional smartphone engagement among specific academic populations like language students. Despite growing research on college students globally, the unique digital behaviors of language learners, shaped by their reliance on mobile applications (MAs) for vocabulary acquisition and communication, remain underexplored. This study aimed to address this gap by investigating MA usage patterns among 31 third-year English majors at a Chinese university, all perceived to be addicted phone users based on the Smartphone Addiction Scale-Short Version (SAS-SV), to examine correlations with academic performance, personality traits, and addiction perceptions, thereby contributing to a nuanced understanding of smartphone use in this context.

Key findings revealed that 150,551 minutes (2,509 hours) were spent on the top three most frequented applications (WeChat, Xiaohongshu, and Douyin), peaking at 1,291 hours in Week 1 and 1,217 hours in Week 2. Despite this, 83.9% denied addiction, attributing use to productivity and control, while 16.1% admitted compulsive behaviors impacting sleep and academics. No significant correlations emerged between MA usage time (MAUT) and academic performance or personality traits, though extraversion was associated with greater MAUT. Among the Big-Five traits, only Conscientiousness showed a strong negative relationship with objective usage time (r = −.806, p < .001); extraversion, neuroticism, openness, and agreeableness were unrelated. Qualitative data consistently highlighted seven functional motives (1) communication, (2) language study, (3) entertainment, (4) information seeking, (5) social networking, (6) practical tools, and (7) health tracking – reinforcing the predominantly functional nature of high engagement in this cohort.

These findings demonstrate that smartphone “addiction” in China is not monolithic but profoundly shaped by academic discipline, platform ecology (WeChat’s “everything-app” role), and cultural definitions of functionality. In language majors, very high but largely productive smartphone use appears to represent an adaptive academic strategy rather than pathology, directly challenging the universal application of current addiction scales to pedagogically digital-native disciplines.

Practically, blanket screen-time restrictions are inappropriate for language students. Universities should instead adopt discipline-specific digital wellness policies that embrace functional high engagement, mandate evidence-based MALL integration (Duolingo, Shanbei, Quizlet) into curricula and assessments, teach self-regulation and attention-management skills from Year 1, and reserve targeted counselling for the minority who exhibit clear compulsive symptoms. Leveraging WeChat mini-programs for digital-literacy training may prove especially effective in China’s unique ecosystem.

Ultimately, the results underscore the urgent need for context-sensitive, objective, and culturally grounded approaches to smartphone research, policy, and pedagogy in Chinese higher education, ensuring technology’s transformative benefits for language learning are harnessed rather than stigmatised.

## 7. Limitations

The study has several limitations that should be considered. First, its small sample of 31 third-year English majors (predominantly female) from a single Chinese university limits generalizability to broader populations or other academic contexts and may overlook cultural or institutional variations. Second, the two-week data collection period, while controlled, may not capture long-term usage patterns or seasonal fluctuations, restricting insights into sustained addiction trends [6]. Third, despite using objective app-tracking data, the reliance on self-reported perceptions of addiction introduces potential bias, as students may underreport compulsive behaviors due to stigma or lack of awareness. Fourth, the study did not account for external factors, e.g., social pressures or mental health conditions, that could influence MA usage, a gap noted in prior research [1]. Fifth, the focus on self-identified addicted users may skew findings toward higher usage, potentially missing moderate users’ experiences. Finally, though the study was introduced to participants as a study on mobile phone usage, rather than addiction, it is important to note that the SAS-SV items inherently probe compulsive behaviors (e.g., loss of control, withdrawal), which could subtly cue participants to frame their usage in justificatory terms during daily notes and 150-word reports, potentially aligning with impression-management tendencies in stigmatized topics. Future studies could further mitigate this by using fully anonymized or delayed screening, or employing neutral-item questionnaires to isolate framing effects.
